# The Lived Experience of Persons with Spinal Cord Injuries Engaging in Remote Work: A South African Perspective

**DOI:** 10.1155/2023/8671123

**Published:** 2023-10-24

**Authors:** Alicia Swart, Jerome Peter Fredericks, Lee-Ann Juliana Jacobs-Nzuzi Khuabi

**Affiliations:** Division of Occupational Therapy, Stellenbosch University, Cape Town 7500, South Africa

## Abstract

In South Africa, traumatic SCI cases are disproportionately high in comparison to other countries. Low retention and maintenance of work for persons with SCIs are further exasperated by structural barriers and societal stigma. Persons with SCIs have the ability to contribute to the labour market, and doing so could lead to the improvement of their quality of life and socioeconomic status. Addressing engagement in work as a meaningful occupation for persons with SCIs is essential to mitigate occupational risk factor and uphold occupational justice. Currently, there is a paucity of literature on remote work practices for persons with SCIs. This study seeks to add to this body of knowledge to increase occupational therapists' awareness of the possibility of remote work to facilitate the inclusion of persons with SCIs in the open labour market. This phenomenological study explored the facilitators and barriers of engaging in remote work in the open labour market for paid employment, as experienced by persons with SCI within the Gauteng metropole, South Africa. Data was collected via semistructured interviews from four participants and analysed thematically. Four themes were generated from the data: (1) intrinsic facilitators of remote work, (2) extrinsic facilitators of remote work, (3) intrinsic barriers to remote work, and (4) extrinsic barriers to remote work. Remote work can be utilised to include persons with SCIs in their chosen occupation of work and is a holistic and client-centred approach. Remote work should not be seen as the sole method of inclusion but can also be used in conjunction with traditional office work to accommodate persons with SCIs in the workplace.

## 1. Introduction

The World Health Organization (WHO) [1] estimates that globally, 250 000 to 500 000 people sustain spinal cord injuries (SCI) per annum, further estimating that up to 90% of those incidences are due to traumatic causes. It is estimated that in South Africa, 75.6 people per million are persons with SCIs [2] with 61.1 people per million accounting for persons with traumatic SCIs [3]. This rate of traumatic SCI cases may be attributed to the high occurrence of motor vehicle accidents and violent events such as gunshots and stab wounds within the South African context [2]. Furthermore, the mean age of persons with SCIs is between 21 and 30 years of age, those who would typically form part of the productive workforce [3]. The sudden onset of SCI due to a traumatic event often leads to a disruption in employment [4]. The disruption in immediate employment not only disrupts the flow of income for these individuals, but evidence shows that the return and maintenance of employment for persons with SCI remain low [5]. This has a vast economic impact on the income and wealth of persons with SCIs. Low retention and maintenance of work for persons with SCIs are further exasperated by structural barriers and societal stigma [6].

Addressing engagement in work as a meaningful occupation for persons with SCIs is essential. Persons with SCIs have the ability to contribute to the labour market, and in doing so, it could lead to improved quality of life and improved socioeconomic status by being afforded the opportunity to earn an income [7]. The United Nations Convention on the Rights of Disabled People [8] indicates that persons with disabilities have the right to engage in work activities on an equitable level. Despite this, many persons with SCIs have lower rates of employment [6]. The South African Human Rights Commission (SAHRC) has indicated that the low labour market absorption of persons with disabilities in South Africa remains problematic [9].

Nonetheless, persons with SCIs are not the only people that have difficulty with employment within South Africa. Stats SA [2] indicates that 30.1% of persons living in South Africa are unemployed. The high unemployment rate in South Africa results in poverty, stigma, and social division [7]. Even so, not all South Africans are considered equal in the quest for employment. According to Eide and Loeb [10], statistics show that in developing countries, persons with disabilities are only employed at a rate of two out of ten, with South African statistics showing only 1.8% of persons with disabilities are employed [6]. These statistics reflect that person with disabilities, such as those with SCIs, have high unemployment rates, and even if they are employed, the retention of employment remains low.

Occupational therapists are typically involved in facilitating the work engagement of persons with disabilities such as persons with SCIs [11]. Occupational therapy intervention would include the recommendation of reasonable accommodations such as assistive devices and workplace adjustments to improve engagement in work [11]. The Department of Public Service and Administration (DPSA) policy on reasonable accommodation and assistive devices for employees with disabilities in the public service [12] states that reasonable accommodation in South Africa has not been adequately integrated with performance management systems of employees, employee wellness programs, or occupational health and safety of the employee. DPSA indicates that reasonable accommodations often only occur on an ad hoc basis, focused on physical and communication barriers. While the South African Employment Equity act of 1996 indicates that no person may be discriminated against based on disability, this includes indirect and direct discrimination. Therefore, persons with disabilities such as SCI should be provided with reasonable accommodations that assist them in fully participating in their work [12].

Employers should implement these accommodations as a means to uphold persons with disabilities' right to work as stated in Employment Equity Act 55 of 1998 and the Code of Good Practice on Employment of Persons with Disabilities [13]. However, this is often challenging, given that within the open labour market, persons with disabilities face numerous barriers due to the rigidity of workplace structures and the lack of adjustments [14]. As the fourth industrial revolution looms, occupational therapists must seek alternatives to traditional work and work environments [14]. The fourth industrial revolution has shifted the focus from in-person work occupations to technologically driven initiatives with overwhelmingly more virtual engagements [15]. This shift in work engagement warrants the exploration of alternatives such as remote work. The argument for such alternatives is strengthened by the global COVID-19 pandemic where there has been a shift in the labour force from venue-based employment to remote and virtual work [16].

Currently, there is a paucity of literature on remote work practices for persons with SCIs. This study, therefore, seeks to add to this body of knowledge to increase occupational therapists' awareness of the possibility of remote work as an option to facilitate the inclusion of persons with SCI in the open labour market. As little is known about those factors affecting persons with SCIs' engagement in remote work within the open labour market, this study seeks to explore the lived experiences of persons with SCIs who are currently or have been engaged in remote work within the open labour market.

The purpose of this study was to gain an increased understanding of the barriers and facilitators of engaging in remote work in the open labour market, for paid employment, for persons with traumatic SCIs from the perspective of those (i.e., persons with SCIs in remote employment) who have lived experience of this phenomenon. The insight gained can hopefully guide occupational therapists regarding the skills training and vocational preparation that is required to facilitate the employment of persons with SCI. It could further inform occupational therapists about the recommendations they make to employers regarding the reasonable accommodations needed to enable persons with SCIs and persons with other disabilities alike, to engage in work and thereby increasing the likelihood that persons with SCIs can continue to make a meaningful contribution to the labour force while engaging in work from home. An improved understanding of the lived experience of persons with SCIs engaging in remote work could inform the development of employment retention strategies for persons with SCIs who are currently employed in the open labour market. Given the paucity of literature available on remote work practices for persons with SCIs within developing contexts, this study seeks to provide a South African perspective on the facilitators and barriers of engaging in remote work in the open labour market for paid employment, as experienced by persons with SCIs.

## 2. Methodology

### 2.1. Research Design

The research was situated in an interpretivist paradigm which allowed the researcher to explore the barriers and facilitators to remote work, by interpreting the lived experiences of the participants [17].

Qualitative research was undertaken as it provided a deeper understanding of the participants' lived experiences of the research phenomenon [17]. The experiences, according to Kielhofner, can only be explained by the participants themselves as they are seen as experts in the field [18].

A phenomenological research tradition was employed as it focuses on participants' lived experiences of the studied phenomenon [19]. By exploring the lived experiences of the participants, the researcher was able to identify the barriers and facilitators as experienced by persons with SCIs who are engaging in remote work within the open labour market.

### 2.2. Study Population

The study population included persons with traumatic SCI who have engaged/are engaging in remote work within the open labour market for remuneration within the Gauteng metropole. Gauteng is divided into three metropolitan municipalities: the City of Johannesburg, the City of Ekurhuleni, and the City of Tshwane Metropolitan Municipalities.

### 2.3. Study Sampling

Participants were selected using purposive sampling (see Table 1 for selection criteria). Purposive sampling is a nonrandom process used to select participants based on specific qualities that they hold and who are information-rich sources who are able to allow for a deeper understanding of the research phenomenon [18]. A snowball recruitment strategy was used. This is a technique employed by using social networks to reach hidden or hard-to-find populations [19]. Social networks included the professional networks of the researcher, community forums, and organisations specific to persons with SCIs.

The envisioned sample size was five participants who would engage in three semistructured interviews. The sample size was, however, determined by data saturation.

Data saturation is the point at which the researcher no longer finds new similarities or differences. However, determining data saturation could prove to be elusive. Guest et al. suggest that the following factors can be used to assist in determining data saturation: (1) the quality of the data, (2) the study's scope, and (3) the nature of the topic.

Utilising these factors in data saturation assisted in gathering sufficient data for a deep description of the researched topic to be drawn. Data saturation was determined once participants were unable to discuss new information related to the barriers and facilitators of remote work. Participants were initially able to describe and discuss their lived experiences of remote work robustly with previous statements leading to new insights into the barriers and facilitators as they experience remote work. As the information was shared throughout the interviews, toward the end of the interviews, it was evident that participants were duplicating information from previous descriptions, finally reaching data saturation. No new information came to light, even with elaboration prompts provided. Therefore, it was determined that data saturation had been reached.

### 2.4. Data Collection Method

Semistructured interviews, with the use of an interview guide (see the appendix), were utilised to gather qualitative data.

### 2.5. Data Collection Procedure

Following approval from the Health Research Ethics Committee (HREC) (X), gatekeepers in the field request access to possible participants that meet the selection criteria.

Potential participants were contacted telephonically by the person referring them to the study to ensure that consent to share their contact details had been given, to comply with POPIA. Language of choice was established during this consent process and relayed to the researcher; all potential participants selected English as their language of choice.

A contact list with contact information for potential participants, as provided by gatekeepers, was created and kept secure on a password-protected computer. The researcher contacted the participants via email. The potential participants were provided with information regarding the research study. The participants had an opportunity to clarify any information provided to them or ask questions as needed. Once all information had been made clear regarding the responsibilities of the researcher and the participant, the participant was able to either provide online informed consent to be included in the study or could opt out of the research study. Online consent or opt-out was recorded on the contact list. Online informed consent was obtained from all participants before commencing the data collection process. An online *Google* form was utilised to gain consent by using a tick box that was submitted before the interview. The participant was provided with three choices as to how interviews could be conducted, namely, face-to-face, telephonic, or virtual video calling via *Microsoft Teams* or *Zoom*. All participants in this study opted to conduct their interviews virtually via *Microsoft Teams* or *Zoom*.

An interview guide (the appendix) was followed, and all interviews were audio-recorded, using the virtual platform's recording option. Each participant was interviewed on three different occasions individually, for 45–60 minutes.

The first interview focused on an explanation of the aim of the study, rapport building, and demographic information gathering. The second and third interviews dealt with collecting the necessary data to answer the research question. The second interview focused on the participant sharing their lived experience of working remotely as a person with an SCI.

Field notes were taken during each interview. The notes were analysed before commencing the next interview. The field notes assisted in illuminating areas that required clarity or further elaboration, in line with the research question.

Should a participant have appeared or expressed distress due to sharing their experience, the participant would have been referred to the relevant gatekeeper with whom they had an established relationship. The gatekeeper would then have, if required, referred the participant to their local health clinic to consult with a mental health professional. No participant in this study, during the data collection, required any such referral.

When data saturation was not reached, participants were asked to identify other persons with SCIs who engage in remote work in the open labour market and who could be contacted and invited to participate in the study (snowballing). Participants contacted potential other participants to gain consent to share their contact details with the researcher. During the snowballing recruitment, the contact list was utilised to record any other participants' contact details.

Overall, 20 potential participants were contacted. One potential participant did not meet the inclusion criteria upon further conversation. Four participants were able to arrange interview times. The 15 other potential participants did not respond to the initial or follow-up emails inviting them to participate in this study. Once new participants had been identified, the same process of informed consent and data collection was followed as outlined above.

To enhance trustworthiness and rigor, Lincoln and Guba's model of trustworthiness and rigor for qualitative research was utilised. The following criteria were used to ensure that the research would be trustworthy: (1) credibility, (2) transferability, (3) dependability, and (4) confirmability.

Credibility was enhanced in this research by peer examining, as the research supervisors guided the process to illuminate and address any researcher bias. Member checking was also employed by the researcher. Since multiple participants' experiences were used, the data collected was collated to ensure the enhancement of credibility. The researcher employed multiple strategies to ensure that transferability was strengthened. Thick descriptions of the participant's experiences, context, and theoretical and methodological approaches were completed to assure that these lived experiences of the participants were unambiguous. An audit trail ensured the enhancement of dependability. The audit trail consisted of peer examination and thick descriptions of the methodology and theoretical context. In using these techniques, the researcher was able to ensure that data collected over time was analysed, collected, and viewed in the way it was set out initially. In this research, confirmability was ensured by using quotes of participants' responses. The researcher made use of journaling to ensure that reflexivity was consistent throughout the research process, to identify any biases.

### 2.6. Data Analysis

The data was analysed using the Braun and Clarke six-step thematic analysis [21]. Thematic analysis is a method used to interpret meanings and patterns within qualitative research [22]. It was used in this study to interpret meaning from the lived experiences of persons with SCIs working remotely, looking for patterns that facilitated engagement in remote work or posed barriers. This method had the benefit of being flexible in sample size, research question, and approach [21]. Moreover, it provided an accessible and systematic manner in which to produce codes and themes from the data gathered [21]. The dataset consisted of the transcribed interviews and field notes. The researcher continued to familiarise herself with the dataset by rereading transcripts and field notes.

Thematic analysis was applied to the study as follows:

#### 2.6.1. Phase 1: Familiarising Yourself with the Data

According to Braun and Clark, the researcher is required to immerse herself in the data. This consisted of reading field notes and listening to audio recordings of the interviews conducted.

The field notes were analysed during the data collection phase, before the next interview with the participant and indicating areas to discuss further. Additional notes were made while listening to the audio recordings of all the interviews. An analytical approach to listening was taken to create meaning from the data. The researcher transcribed the data from the interviews; those interviews completed in English and Afrikaans were transcribed into their respective languages. The analysis of this data occurred in the language of the interview; interviews conducted in Zulu would have been transcribed by a Zulu-speaking transcriber and, thereafter, translated into English, if needed. However, all participants in this study were proficient in English and selected this as the language of their choice, and therefore, a translator was not utilised.

#### 2.6.2. Phase 2: Generating Initial Codes

Systematic analysis commenced with the generation of codes per the interview transcriptions. The codes served as the smaller components which later generated themes. Codes were generated by thoroughly reading through transcripts and identifying similarities in topics discussed by the participants. Thematic analysis allowed for making correlations within data and across data. The correlation process commenced during the coding process.

Coding was completed using semantic and latent coding approaches. Semantic codes in this study produced codes as lived experiences expressed by the participants. While the researcher produced latent codes, deeper interpretation of the lived experiences was achieved, based on the transcriptions and field notes. As recommended by Braun and Clark, one interview was coded before the next in this study. All codes were seen as part of the research question's answer.

#### 2.6.3. Phase 3: Searching for Themes

The researcher followed the process of theme identification to identify patterns in the data collected. These patterns emerged from a combination of codes. Themes were derived from the collation of codes, as identified from the interview transcription. Active engagement with codes was essential, as theme searching was only successful if the researcher applied herself to the data available. Braun and Clark describe this process as the construction of themes rather than the discovery of themes.

Within the research study, the codes were reviewed and the researcher constructed themes from patterns that had been noticed between codes. The themes provided order to the raw data.

#### 2.6.4. Phase 4: Reviewing Potential Themes

The process of reviewing themes was particularly important as it was a way for the researcher to monitor the quality of the themes. A process of matching themes with extracts from the dataset was conducted, to establish whether the data matched. If the data extract and theme did not match, the researcher needed to reorganise or discard themes. This process was done to ensure that the researcher's themes were relevant and in line with the lived experiences as expressed by the participants.

This process was guided by questions of relevance. Braun and Clark suggest that the researcher set boundaries for themes to ensure that the inclusion and exclusion of a theme were known. They also suggest weighing whether the theme held value in answering the research questions. The researcher ensured that all themes related directly to the barriers and facilitators as experienced by persons with SCIs engaging in remote work within the open labour market for remuneration. In that way, the researcher was able to determine the relevance of a theme.

#### 2.6.5. Phase 5: Defining and Naming Themes

The researcher has to define and name themes, to summarise the essence of each theme. Themes that share relevance, prevent duplication, and directly relate to the research questions are seen as good thematic analysis.

The process of defining and naming themes creates the opportunity for the researcher to fine-tune the analysis and to shape the analysis into a narrative of the data, to create a vivid understanding of the lived experiences of persons with SCIs engaging in remote work.

The researcher ensured that the identified themes were defined and named, linking directly to the barriers and facilitators as experienced by persons with SCIs doing remote work.

#### 2.6.6. Phase 6: Producing the Report

The phase of producing a report within qualitative data was an ongoing process. Writing up the data was continuous and occurred parallel to the analysis process. The focus of this phase correlated directly with Phase 5, as it was concerned with providing a clear description of the data. The researcher aimed to provide the reader with a concise and compelling argument for the research question which is an authentic representation of the participants' lived experiences of engaging in remote work within the open labour market, for remuneration.

## 3. Findings

The data was collected via virtual semistructured interviews with four participants (see Table 2 for the demographic profile of the participants). The data were collected over 15 hours, comprising of two to three one-hour interviews per participant, followed by member checking.

### 3.1. Presentation of the Findings

Four themes were generated from the data, i.e., (1) intrinsic facilitators of remote work, (2) extrinsic facilitators of remote work, (3) intrinsic barriers of remote work, and (4) extrinsic barriers of remote work. As there is no one correct way to depict the findings in a qualitative study [23], the findings will be depicted in the format of a table (see Table 3). The choice of a table is further supported by authors such as Cloutier and Ravasi [24]. The themes and subthemes are reflected with the associated illustrative quotes. To allow the reader to make sense of the data (i.e., follow the story), descriptors of each of the themes and subthemes have been included.

## 4. Discussion

The discussion of the study's findings will be situated within the field of occupational science. Wilcock [25] described humans as occupational beings with the belief that occupation is essential to sustained being and quality of life. She indicates that exclusion from occupations due to barriers has inherent risks to a person's quality of life. These barriers can include environmental and access barriers, negative attitudes of persons regarding persons with SCI, or a lack of support [26, 27]. It is imperative to view the occupation of work as meaningful to a person as it provides inclusion in social and work structures and provides a sense of identity and access to socioeconomic gain [24].

An occupational science lens within an occupational justice framework [28] can be used to best conceptualise the barriers and facilitators as experienced by persons with SCIs, engaging in remote work. When we use the occupational risk factors as described by Hitch et al. [28] to discuss the findings of this study, we can understand how remote work either mitigate or reinforces occupational risk factors for persons with SCIs while engaging in remote work.

The occupational justice framework as discussed by Chichaya et al. [29] and Mostafa et al. [30] focuses on ensuring that each individual has an occupational choice in areas such as work. The individual is able to engage in their chosen occupation of work in an occupationally just environment, free from barriers, promoting occupational performance. An environmentally just environment encompasses the physical environment as well as the psychosocial environment the person works in. The occupational justice framework is aimed at looking at occupational injustices analytically, to understand factors affecting occupational justice. Therefore, having a focused approach to mitigate these injustices [29]. Occupational justice is described as a combination of occupational risk factors as discussed below.

Occupational alienation concerning work for persons with SCIs can relate to the lack of reintegration into the occupation of work post-SCI [26, 28, 31]. This can be due to a lack of environmental adaptations, a lack of knowledge and attitude from employers and employees, or insufficient knowledge and access to reasonable accommodation options. Remote work can assist in diminishing the risk of occupational alienation by providing employees with SCIs the opportunity to engage in remote work options on an individual level to suit their health and functional needs. This is reflected in theme 1 (subtheme 1.1) of this study. Participants found that a tailored approach to work is possible while engaging in remote work, therefore decreasing their risk of occupational alienation. They were able to actively engage in work as they were able to schedule their time and arrange their environment to suit their individual needs, and an occupationally just environment was upheld. Moreover, theme 2 further illustrated that through overcoming geographical boundaries by the use of remote work, participants had increased access to work opportunities further decreasing the risk of occupational alienation.

Occupational deprivation refers to a person being prevented from engaging in their chosen occupation [26, 28]. Previously, remote work options and technologies were not as readily available for persons with SCIs, preventing them from engaging in their chosen occupation of work. Such technologies are becoming less costly and more readily available serving as an important tool for reasonable accommodation. While some job duties remain [30] difficult or impractical to translate into remote work as discussed in theme 3 (subtheme 3.1), remote work has allowed many employees to continue engaging in work while working remotely. This is illustrated again in theme 1 of this study. Participants were able to create a work environment, while working remotely, which suited their needs, enabling them to continually engage in work. Remote work is facilitated by improved technology and the improved knowledge and skills of employees and employers, as reflected by theme 2, where participants indicated that they felt remote work is the future of employment due to technological advancements that make remote work possible. Therefore, remote work can decrease occupational deprivation by providing persons with SCI the opportunity to engage in work more readily.

Similarly, remote work can indirectly relate to occupational deprivation of social engagement in the workplace as well as deprivation of the role as an in-office employee, leading to possible marginalisation.

Thus, occupational marginalisation is the employee being continually excluded from occupations like work-related social interactions [26, 28, 32]. Participants in this study found decreased social interaction to be a barrier to remote work, as reflected in theme 3. Additionally, should a person continually find difficulty in entering the labour force due to their SCI, the person may also experience occupational deprivation related to the occupation of work. A person-centred approach to purposeful occupation is imperative when viewing barriers and facilitators of work and remote work options.

Persons with SCIs who are not integrated into the labour force due to their SCI can experience occupational imbalance as a result of occupational injustices. They are excluded from the workforce and therefore only engage in limited occupations. Furthermore, exclusion from employment affected persons with SCI socioeconomic status, often resulting in further social injustices [6].

Remote work options can assist in mitigating the exclusion from the workforce for persons with SCIs, and occupational imbalance can be prevented. Participants in this study found that economic opportunities were more readily available as geographical boundaries were overcome (see theme 2). However, the opposite can also be true, persons who engage in remote work with limited work-related boundaries can be at risk of disproportionally engaging in work above other chosen occupations such as family time or leisure. Participants reflected that when working remotely, employees should be able to manage a good work-life balance as indicated in theme 1.

Therefore, the discussion is focused on understanding how occupational justice can be upheld. Whether remote work options can mitigate occupational injustices and serve as a facilitator of remote work or if occupational injustices are more likely to occur during remote work serves as a barrier to remote work.

This chapter will discuss the four major themes that were generated, focusing on how these themes impact occupational risk factors for persons with SCIs.

### 4.1. Facilitators

A facilitator as defined by the WHO [29] is a factor, environment, or person that has a positive influence on an outcome or increases an individual's performance capacity and participation.

To discuss the findings of this study, a facilitator will reflect on (1) how the engagement in remote work improved the participant's engagement in the work itself and (2) how remote work engagement has improved the outcome of work performance for the participant.

#### 4.1.1. Theme One: Intrinsic Facilitators

Intrinsic facilitators of remote work categorise facilitators of remote work into that which is intrinsically assisting the participant in participating effectively in work. Merriam-Webster defined the word “intrinsic” [33] as the essential nature of or the constitution of a specific thing. The theme of intrinsic facilitators, therefore, refers to the essential nature of the job itself and the essential nature of the employee with an SCI that assists with the successful participation in his or her work duties.

*(1) Subtheme 1.1: Remote Work Promotes a Tailored Approach to Work Engagement*. Participants expressed that because of the nature of remote work, they were able to make the necessary adaptations to their work schedules and environment, on an individual level, to best suit the management of their needs and limitations. One such adaptation a participant made was to lay out his remote office to include a desk suited to his wheelchair height. Remote work allowed for an increased sense of autonomy, flexibility, productivity, and easier management of the secondary health complications of SCI.

Participants indicated that working remotely facilitated their work participation and found they were more time efficient since they spent less time commuting to and from work. They also found they were more productive, as they experienced fewer interruptions and distractions during the day. A study conducted in 2021 [34] found that employees engaging in remote work experienced increased productivity as well as self-efficiency and autonomy. Furthermore, Bloom et al. [35] found that persons working from home (WFH) experienced a 13% increase in productivity as well as improved time efficiency and schedule flexibility.

Furthermore, a 2015 systematic review [33] found that one of the key facilitators for workplace accommodations for persons with disabilities is flexibility in their work schedules. It is evident from the literature and participants' experiences that remote work affords persons with SCIs the opportunity to work more effectively and with improved flexibility.

Participants in the current study indicated that they experienced improved physical and mental wellness while working remotely as they had autonomy over their work schedules, affording them the ability to engage in other activities of their choice while still being able to complete their job tasks effectively. These experiences echoed previous literature regarding remote work [34]. This flexibility assisted participants in maintaining occupational balance by engaging in a range of chosen occupations while working remotely. In this way, remote work flexibility enhances occupational balance and decreases the risk of occupational imbalance.

Remote work creates a tailored work approach for participants. This tailored approach extended beyond their work productivity improvement to excluding access barriers from their daily work engagement.

Participants reported that they were able to engage freely in their work duties without needing to overcome or consider access barriers that would impact their work participation success. Occupational marginalisation is inevitable because access barriers create continued difficulties in the workplace that hampered work success for employees with SCIs. In a study in 2018, Dorsett and McLennan [36] found that persons with disabilities returning to work experienced that access barriers in the workplace were one of the main limitations to successfully engaging in work tasks. Kaplan et al. [37] suggest that remote work can be negotiated as a reasonable accommodation for persons with disabilities to overcome barriers to successful work participation. They suggest a framework where trust and credibility as well as organisational policy are the guiding pillars. Furthermore, McNamara and Mason Stanch [38] found that after the mandated rules during the 2020 COVID-19 lockdown, accommodations that would previously have been seen as special requests were now deemed routine, opening doors for persons with disabilities to access work more readily [39]. Participants in this study were able to engage in their chosen occupation of work remotely, without barriers. In this way, remote work facilitated occupational justice and mitigated occupational marginalisation.

Participants further indicated that they were able to engage in work more effectively when working remotely because they were able to manage secondary complications such as their bladder and bowel routines more effectively, indicating they had access to toilets more readily when working from home.

It is clear from this subtheme that participants experienced improved ability to engage in their job tasks more successfully and more effectively due to the tailored approach that remote work provides.

The occupational therapist must consider the use of remote work for persons with SCIs when in the open labour market. By so doing, occupational justice will be upheld, assisting with the employment of persons with SCIs in the open labour market with improved mitigation for functional impairments.

These intrinsic facilitators improved the overall work experience of the participants.

*(2) Subtheme 1.2: Remote Work Requires Positive Personal Attributes*. Participants experienced that when engaging in remote work, they required positive personal attributes such as self-discipline, accountability, and the ability to maintain a good work-life balance. Participants experienced that these attributes surpassed good work habits as they felt this was required more prominently when they commenced remote work.

These positive attributes are described in the remote work literature [34–36] and include being able to maintain a good work-life balance [36], good time management skills, and accountability [35]. The literature suggests that having these attributes assist in the success of the participation in remote work.

Participants discussed that when they realised the importance of good time management skills when working remotely, they were more successful in their job tasks. Improved time management also helped participants maintain a better work-life balance. One participant discussed how boundaries in his home such as work times and leisure times assisted with maintaining a good work-life balance. Another participant indicated that he experienced being more successful in remote work when he worked in his home office because he was more focused as opposed to working in the lounge.

Participants report that both time and environmental boundaries were important for them to maintain these positive personal attributes to engage in remote work successfully. One participant discussed that being accountable was a highly valued attribute, as he was able to schedule his workdays to assist with engagement in leisure. However, if he did not have a sense of accountability to meet his job demands, it would have been easy not to engage successfully with his work. Therefore, by possessing these attributes, the employee could continue in his chosen occupation of work, decrease the risk of occupational marginalisation, and uphold occupational justice.

Understanding these personal positive attributes can assist the occupational therapist, employer, and employee negotiation strategies when discussing remote work as either a natural progression into technological advancements or as a reasonable accommodation [35].

#### 4.1.2. Theme Two: Extrinsic Facilitators of Remote Work

Merriam-Webster [33] defines extrinsic as originating from outside or originating outside and acting upon a part or a whole.

An extrinsic facilitator refers to what is outside of the job itself and what is outside of the employee that it acts upon, to assist the employee with an SCI with participation in his or her work duties.

This theme reflected that to make remote work successful, extrinsic facilitators, environmental or personal, that positively influence the employee's performance capacity and participation must be implemented. These extrinsic facilitators illustrate that remote work enlarged the geographical area where work was available. Moreover, participants experienced that when they had the necessary infrastructure required to work remotely, it aided in the success of engaging in work duties remotely. This ensured that work duties could be completed as if the employee was in the office environment.

*(1) Subtheme 2.1: Remote Work Promotes Geographical Access and Diversity*. Remote work provided participants with flexibility in time but also in geographical location. Remote work facilitates increased opportunities for work, irrespective of geographical boundaries. Participants found that with remote work, they were able to decide more freely where their homes would be situated and whether they will work from home or a holiday destination. Participants also indicated that they were more exposed to global opportunities for work, indicating that remote work promoted diversity in workplaces.

A 2020 article [37] refers to the concept of work from anywhere (WFA) rather than work from home. The concept of WFA is more closely related to the definition used in this study for remote work, referring to working from any location suited to completing the job tasks. Choudhury et al. [40] indicated that flexibility in geographical location was an added benefit to employees engaging in WFA programs. Mulki et al. [41] further emphasise that remote work provides a global scale for work engagement. This correlates with the experiences of participants in this study, indicating that since working remotely, they have had the opportunity to work in global teams, expanding cultural diversity in the workforce.

Overcoming geographical barriers can assist with persons with SCIs' employment within South Africa, to uphold occupational justice. The national unemployment rate has a dire impact on all areas of employment. However, with remote work options, persons with disabilities can be introduced to a wider area of employment opportunities and, in this way, overcome employment restrictions in a geographical area. Moreover, persons with SCIs residing outside cities can engage in work remotely without having to manage commuting to and from a geographical location like an office. Galanti et al. [42] further discuss remote work as providing persons with SCI the opportunity to engage in a wide range of remunerated work in the open labour market.

*(2) Subtheme 2.2: Remote Work as the Future of Employment*. Remote work is not a novel idea in certain sectors but speaks to the future of employment. Through the use and advancement of technology, remote work options are more accessible to employees. With the onset of the COVID-19 pandemic, remote work has become a routine engagement for most employees [35]. Virtual and remote methods have been employed to complete work duties, and this can specifically be used to facilitate the engagement in work for persons with SCIs.

McNamara and Mason Stanch [38] illuminate that throughout the last few years, the advancement in technology has been exponential, providing improved access to a variety of work tasks for persons with disabilities. The participants in this study experienced that throughout engaging in remote work, the ease of use of technology has improved and they foresee technological advancement within their current organisation.

A 2020 article by Dingel and Nieman [43] indicates that 37% of all jobs in the United States of America can be fully conducted remotely. Furthermore, a survey in April 2021 [38] indicated that more than 50% of employees would want to continue working from home more than three days per week. Bartik et al. [34] further indicate that larger companies foresee remaining with some form of remote work postpandemic. Participants echoed this in their experiences, indicating that in their current working environment, a hybrid model will be best suited for work engagement.

Considering how the landscape of employment would look in the future is imperative for the occupational therapist to assess how best to prepare persons with SCIs for engagement in remunerated work in the open labour market. Therefore, considering remote work options for persons with SCIs is not only beneficial but also sustainable.

Through both themes one and two, it is clear that by providing persons with SCI the opportunity to work remotely, occupational justice can be upheld while overcoming occupational marginalisation and occupational alienation. It also allows persons with SCIs to engage in the work of their choice and in the sector of employment they choose. They were able to engage in work more readily as access barriers were mitigated with remote work and had more opportunities to manage secondary health complications. Participants' awareness of positive personal attributes further aided their engagement in remote work. These facilitators all assisted persons with SCIs in being able to engage in their chosen occupation of work more successfully.

The occupational therapist must consider the use of remote work for persons with SCIs as a reasonable accommodation or platform for work in the open labour market.

### 4.2. Barriers

A barrier, as defined by the WHO [44], is a factor whether environmental or personal that has a negative influence on an outcome or restricts an individual's performance capacity and participation.

A barrier, for this study, will reflect how the engagement in remote work restricted the participant's engagement during work and how engagement in remote work has negatively impacted the participant or the outcome of work performance for the participant.

#### 4.2.1. Theme Three: Extrinsic Barriers

As discussed, the word extrinsic refers to what is originating from the outside [45]. Therefore, an extrinsic barrier refers to what is outside of the job itself and what is outside of the employee which acts upon and decreases the employee with an SCI's participation in his or her work duties or negatively impacts the employee.

The extrinsic barriers, as discussed by participants, are that remote work was not accessible to all levels of employees and sectors of employment. Additionally, the success of remote work was affected by the support that the employee with an SCI received from the company and the management team.

*(1) Subtheme 3.1: Employment Level and Sector Affect Employees' Access to Working Remotely*. Participants reported that remote work is not necessarily accessible to all sectors or at all levels of employment. Participants indicated that they experienced that the level of employment affected the work duties and the level of support/supervision that an employee requires. These aspects prevent employees from working remotely.

Although all participants in this study were at mid to higher levels of employment, all participants reflected that they would have found it difficult to integrate into a workplace as a junior employee, while working remotely. They stated that the supervision required and the type of work they were doing would have been difficult to complete remotely.

Participants also mentioned that there are logistical difficulties with all employees working remotely, such as with client-facing job duties. They indicated that this can be mitigated with virtual platforms; however, that is not true for all levels or sectors of employment.

Bartick et al. [34] suggested that companies employing persons with higher levels of education or remuneration were most likely to engage in remote work. Dingel and Neiman [30] also found that in higher-income economies, remote work jobs were more widespread than in lower-income economies. They developed an occupation classification for the types of jobs that can be completed remotely. Within this classification, they categorise any job that requires physical activity, moving objects, or working directly with the public as not suitable for remote work [30]. This classification can be useful when negotiating remote work options for persons with SCIs engaging in employment.

Nonetheless, this classification only considered a fully remote work model and did not consider hybrid models of work, such as performing some tasks within an office as needed while performing other tasks remotely, to accommodate the employee's unique needs. The hybrid model was particularly useful for a participant in the current study; she was required to be client-facing for some aspects of her job. However, because there was no access to suitable toilets at her office, she was unable to spend a full day at the office. After negotiations, the participant indicates that she now completes her job tasks following a hybrid model. Other participants also indicated that a hybrid model of work would be best suited to their needs. This is echoed by [39], indicating that the future holds more opportunities to accommodate persons with disabilities by using a hybrid model.

It is, therefore, important to note that the recommendations for remote work for persons with SCIs are not appropriate for all employees. One must consider the level of support and supervision that the employee will require. A person with an SCI must be integrated into a work environment whether virtual or in person with the same support as other employees at their level of employment. Remote work might overcome access barriers or improve flexibility, but if an employee's level of employment requires more focused support, the employee will likely not succeed with a fully remote work option. Employment of a person with an SCI remains a holistic and tailored approach and should be focused on the needs of the employee, weighing all aspects of the job requirements with all the accommodations required. Additionally, remote work can be approached with a give and take, such as a hybrid model which can overcome some limitations of pure remote work and pure office-bound work. In that way, an employee is not deprived of working, but the justice of occupation can be upheld by mitigating factors to create a tailored work experience with the appropriate reasonable accommodations.

*(2) Subtheme 3.2: Lack of Employer Support Impacting an Employee's Capacity to Work Remotely*. Participants indicated that where employers do not support the employee's individual needs nor understand the employee's functional limitations, negotiating remote work as a reasonable accommodation for persons with SCIs can be challenging.

Participants found that remote work options were hampered by a lack of understanding from the employer. Therefore, if the employer did not understand the need for remote work or was mistrustful of the employee, remote work was unsuccessful. Nevala et al. [39] found that when looking at workplace accommodations for PWD, employer support was imperative to the success of accommodations. Dorsett and McLennan [36] also found that employers who are supportive of employees with disabilities have more sustainable long-term employment outcomes.

Participants in this study discussed that once employers were willing to engage in a conversation regarding the employee's specific needs and form a broader understanding of their limitations, only then could adequate work accommodations could be made. In this way, employers then understood the need for remote work options.

Furthermore, participants indicated that remote work success was negatively affected by an inflexible or micromanagement style. Participants felt that when there was a trusting relationship between employee and employer, remote work was more successful. Some participants also suggested that transformation in management styles was necessary. There should be a shift away from micromanagement styles to more output-driven, performance-based management styles. Mulki et al. [41] found that supportive managers aided in the success of remote work practices.

Considering this barrier, the occupational therapist needs to evaluate how reduced understanding and support from an employer can impact the experience of remote work of an employee with an SCI. The occupational therapist should consider the employer's level of understanding of their limitations. This level of understanding does not have to breach the confidentiality of the employee, however, providing education or facilitating a conversation regarding the specific functional limitation experienced by the employee could assist the employer with understanding the value of remote work as a reasonable accommodation.

It is imperative to consider the confounding factors that impact on remote work success for persons with SCI as overlooking these factors can further increase the occupational risk factors.

#### 4.2.2. Theme Four: Intrinsic Barriers of Remote Work

Participants experienced that intrinsic factors such as a stagnant occupational environment, resulting in a lack of social engagement, proved to be a barrier to the employee's success in working remotely. Participants further perceived that they were less physically active while engaging in remote work.

*(1) Subtheme 4.1: Remote Work Hampers Social Work Engagement*. Participants felt that engaging in remote work decreased their social engagement at work, resulting in a feeling of social isolation. Social isolation was cited as one of the barriers for employees working remotely [38]. Additionally, Dorsett and McLennan [36] found that one of the benefits of engaging in work for a person with an SCI was social engagement. Remote work can pose a barrier to such social engagement. Therefore, it is important to consider using mitigation strategies to overcome social isolation for employees engaging in remote work. Literature [38] discussed the use of social media platforms and virtual communication as a method to reduce the feeling of social isolation amongst employees. Alexander et al. [46] further suggest that regular small team connection events can assist with social inclusion as well as social cohesion.

Participants further indicated that office work provided them with the opportunity to practice social skills in a more structured environment. This is especially true for employees newly diagnosed with an SCI or employees entering the workplace for the first time. The participants felt that working remotely does not afford them this practice.

Moreover, participants reported that remote work can also impede work culture and employee morale. This is reiterated by Donati et al. [47] who state that decreased social engagement in persons working remotely without mitigation strategies in place can lead to experiencing uselessness and hopelessness. One participant in this study indicated that she felt that the benefit of lunch or smoke breaks assisted with workplace morale and improved social cohesion. This was echoed in literature [38] where participants stated that informal communication and hallway conversation aided in workplace social engagement. Mulki et al. [41] suggest that having processes in place to assist employees is imperative for mitigating the risk of social isolation and navigating work-related stressors. They propose programs such as mentoring and the pairing of employees, having some face-to-face workshops, and having scheduled weekly meetings to share formal and informal ideas with colleagues.

Participants further felt that remote work can decrease the visibility of persons with SCIs in the workplace.

Understanding this intrinsic barrier can assist the occupational therapist, employee, and employer with mitigating risks early on in the remote work process. This would include assistance in early upskilling of the employee and employer with appropriate ways to aid overall social inclusion within the organisation when engaging in remote work.

Considering this barrier within an occupational science framework, a person with an SCI who engages in remote work can be at risk of occupation imbalance because an employee might be engaging in the occupation of work disproportionately, resulting in a lack of adequate engagement in other occupations. Moreover, the employee engaging in remote work with an SCI can be at risk of occupational deprivation and deprivation of social interaction.

As noted previously, engaging a person with an SCI in work is a holistic and multifaceted approach. Workplace accommodations should always be considered in conjunction with potential risks. Townsend [26] specifically referred to engagement in one's chosen occupation as the basis for health. Therefore, if a person is disproportionally being placed at risk, alternative workplace accommodations should be considered or mitigating these risks should be priorities at the start of the remote work process.

*(2) Subtheme 4.2: Remote Work Impacts Physical Mobility*. Participants experienced that when working remotely, they had less opportunity for physical mobility, transferring in and out of their wheelchairs less as well as shorter distance wheelchair propulsion. Thus, remote work exacerbated sedentary work.

A 2021 article [41] discusses the benefits of physical mobility for persons with disabilities. The authors found a positive association between increased physical activity with improved psychosocial wellness, cardiorespiratory function, and muscular strength. Furthermore, the WHO [48] guidelines on physical activity [44] highlight the benefits of physical activity during the day including improved quality of life and improved cognition and physical function amongst persons with SCIs.

Participants reported that due to the decreased need for commuting and in-office work, they found themselves transferring in and out of the car and wheelchair less frequently. Participants also indicated that their attire differed during remote work; they were not required to get dressed in tighter clothing like suits. They conveyed that dressing in work attire required more physical strength and mobility.

Participants asserted that they were aware of the importance of physical activity for their health, and almost all participants indicated that they prioritise physical mobility during their day. Participants indicated that having the added benefit of a flexible work schedule enabled them to prioritise physical activity during their day, using other occupations. Some participants prioritise therapies while others prioritise home gyms or leisure activities. Moreover, almost all participants were involved in leisure activities that optimised their physical mobility such as wheelchair rugby or hand cycling. Participants reported that engagement in these leisure activities assisted them greatly in maintaining mental wellbeing and their physical fitness and strength during remote work even if their workday was mostly sedentary.

The participants in this study were able to self-mitigate the risks of decreased physical mobility. The occupational therapist and employee with an SCI need to be proactive with a physical mobility schedule and understand the risks of decreased mobility.

## 5. Recommendations

Further research regarding the functional capacity of persons with SCIs and the effect on remote work engagement is required. Additional research is required to indicate how different sectors of employment can utilise remote work options to ensure equitable employment options. Research regarding remote work not specific to telecommunication and virtual forms of remote work should be explored for persons with SCI, for example, completing work-related tasks from home that are not primarily linked to computer-based work.

## 6. Conclusion

This study set out to explore the lived experiences of the barriers and facilitators of remote work for persons with SCI within the open labour market. The participants experienced that remote work had both intrinsic and extrinsic barriers and facilitators, and although remote work assisted them to engage more successfully in their work tasks, some aspects of remote work proved to be barriers to occupational balance. Moreover, the study examined in what manner remote work can be utilised to include persons with SCI in their chosen occupation of work. Using an individual, holistic, and client-centred approach, remote work should not be seen as the only method of inclusion but can be used in conjunction with traditional office work to accommodate persons with SCIs in the workplace.

### 6.1. Limitations of the Study

Potential limitations should be considered when interpreting the findings of this study:

One such limitation is that this study sample only included English-speaking participants, employed in a corporate setting. This could be a potential limitation to the study since cultural, sectoral, and employment levels were not equally represented, with limited diversity. Moreover, all participants in this study had a tertiary education. This is not adequately representative of the South African population as it may imply that persons with SCIs who do not have access to further education or adequate resources can be excluded from remote work options.

Secondly, the findings of this study are not generalisable and may not be transferable to other persons with SCIs engaging in remote work. This is due to the small sample size of the study, compared to the larger population of persons with SCIs working remotely. It is worth noting that the study is aimed at developing an understanding of the phenomenon rather than generalising.

Additionally, as the interviews were conducted via online platforms, observation of body language and rapport building might have been limited. Although all participants reported online platforms to be the most convenient, face-to-face interviews could have assisted in nonverbal communication. Nonverbal communication cues can assist in providing contextual cues or affirm verbal communication for the researcher, thereby providing a more holistic understanding of the participant's experience.

## Figures and Tables

**Table 1:** Selection criteria of the participants.

Selection criteria	Motivation
Persons with traumatic SCIs who are of working age.	The research is aimed at exploring the lived experiences of persons with traumatic SCIs who have worked/are working remotely.
Currently or previously employed within remote work for at least three months.	The participant should have lived experience of working remotely in the open labour market to share their perspectives on the barriers and facilitators of remote work. To do so, they would have needed to be employed in that setting for the universal employment trial period of three months [20].
Speak and understand English, Afrikaans, or Zulu.	The three languages predominantly spoken in Gauteng.
Resides within the Gauteng metropole.	Due to the scope, time, and financial considerations of the research, participants had to reside in the Gauteng metropole.

Exclusion criteria	Motivation
Persons with premorbid psychiatric disorders.	Premorbid psychiatric disorders could be a factor impeding the person's ability to succeed in employment, and this study specifically focused on persons with spinal cord injuries.

**Table 2:** Demographic profile of the participants.

Participant pseudonym	Age	Gender	Educational level	Employment type	Level of SCI	Duration of remote work
1	35	Female	Tertiary education	Corporate	Incomplete T8/T9 (paraplegia)	>2 years; hybrid model
2	Undisclosed	Male	Tertiary education	Corporate	Incomplete C6 (quadriplegia)	Full time as of 2020
3	40	Male	Tertiary education	Corporate	Incomplete C6	Full time as of 2020. Intermittently before 2020
4	Undisclosed	Male	Tertiary education	Corporate	Complete T2/T3 (paraplegia)	Full time as of 2020

**Table 3:** Findings: themes, subthemes, and quotations.

Theme one: intrinsic facilitators of remote work The participants experienced that there are intrinsic facilitators to remote work. These are facilitators that are essential to the nature of the job or the employee with SCI. Participants indicated that remote work engagement facilitated their work engagement. Participants found that when they worked remotely, they were able to tailor their work schedule and work tasks more autonomously. This provided more flexibility during their day to manage their health and wellness. Participants found that remote work tailored their workday in such a way that they were more productive and experienced improved wellness. Participants also expressed that when working remotely, access barriers were excluded from their daily work engagement. Moreover, participants indicated that with a tailored work approach, they were required to manage their day autonomously requiring self-discipline, accountability, and the ability to maintain work-life balance. These intrinsic facilitators assisted with the successful engagement in their work	
Subtheme	Quotation
*Remote work promotes a tailored approach to work engagement* Remote work, as experienced by participants, facilitated accommodations in each employee's work schedule and environment. Access barriers were eliminated during remote work as employees were able to tailor their environment to their individual needs. This allowed employees the freedom to manage their workdays to suit their individual requirements. Remote work allows for an increased sense of autonomy, flexibility, productivity, and easier management of health-related factors due to SCI	Experience of increased flexibility and autonomy in choosing where they work: “I do not spend that much time like sitting in the office … employer is very reasonable in terms of where I work, ‘cause I can essentially… can work from home.” (Participant 1, line 87) “[Remote work is] a lot more flexible. And caters for a lot of different people.” (Participant 3, lines 662–664) This participant reflected on accessibility during in-office work and stated that although some companies are aware of universal access requirements, not all areas are accessible. During remote work, employees were able to tailor their environment to their individual needs: “... all the companies do, make all the provisions you know for wheelchair access and all that. It's not always that cool. You know, sometimes you get to a desk and the desk I cannot get under them, you know? So I'm sitting with my laptops over there and by the end of the day…your shoulders are so tired you...” (Participant 4, Line 83-85 0401) Improved management of health-related factors at home: “[For my] toilet like routine… the one thing … [that] has had the biggest impact … working from home … you do not even think about it.” (Participant 4, lines 96–98) Improved productivity: “I found [I] am a lot more productive …” (Participant 4, line 109)

*Remote work requires positive personal attributes* Participants reflected that remote work requires self-discipline, accountability, and the ability to maintain work-life balance. Employees were able to manage their own work schedules more readily, and therefore, the onus of ensuring their work is completed without direct oversight, rested on the employee. Therefore, employees were required to be accountable and have the self-discipline to meet their job demands daily	Self-discipline is required: “… working from home… not everybody can do it. Um, because it needs a certain discipline and trust as well.” (Participant 1, line 88) “have a little bit of flexibility, but ultimately you know you need to deliver on your job” (Participant 3, line 578) Accountability of the employees is important: “… you commit to a deadline, you need to make sure that you are going to deliver and you need to find a routine” (Participant 3, line 690) Maintaining work-life balance: “I could do cool stuff as well and if I work, you know effectively during working times can also play more.” (Participant 4, line 109)

Theme two: extrinsic facilitators of remote work The participants experienced that remote work provided the option of working outside geographical boundaries. They found this to be an extrinsic facilitator, having increased access to employment opportunities outside their immediate geographical area. Participants further experienced that remote work can become the future of employment given technological advancements and the remote work success they have experienced	
*Remote work promotes geographical access and diversity* Due to virtual option flexibility, remote work facilitates increased opportunities for work, irrespective of geographical boundaries, and has the potential to promote diversity in workplaces	Remote work can overcome geographical boundaries, thereby improving the accessibility of persons with SCI to enter a chosen work sector even if it is not accessible for a person in their geographical area: “… more people that were not usually accessible are now accessible, so cross provincial, international…” (Participant 1, line 348) Remote work provides opportunities for increasing diversity in the workplace: “Our team is now global and we do not work just with our immediate team anymore.” (Participant 3, line 1116)

*Remote work as the future of employment* Although not novel in all sectors, remote work speaks to the future of employment using technology and virtual and remote methods to complete work duties, and this can specifically be used to facilitate the engagement in work for persons with SCIs	Remote work is not novel in all sectors: “… if you look at it from people who are running multinational businesses across the world, this [remote work] has been their norm.” (Participant 1, line 336) “…working in a global role, so they have been OK for years with working and set up at home” (Participant 4, line 196) “… the whole company was introduced to work from home.” (Participant 3, line 286) Working remotely for the foreseeable future, envisioning the future of employment as remote work: This participant indicated that when he invasions his future as an employee, he sees himself working remotely: “to work at home indefinitely” (Participant 2, line 1377) “… all these technologies as well… having things that look a lot more real. … they investing a lot of money in virtual reality” (Participant 4, line 171)

Theme three: extrinsic barriers of remote work Reflects the extrinsic barriers of remote work as experienced by participants. These extrinsic barriers indicated that remote work was not accessible to all employees and sectors of employment. Furthermore, the success of remote work was also affected by the support that an employee with an SCI received from the company as well as the management team	
*Employment level and sector affect employees' access to working remotely* Reflects that remote work is not accessible to all sectors, nor all levels of employment. Employment levels affect the work duties and the level of support/supervision an employee requires. These aspects prevent employees from working remotely	“… the nature of my work could not allow me to be remote [worker].” (Participant 1, line 1463) “[Remote work] depending on the type of work you are doing.” (Participant 4, line 198) “The level that I'm at it is easier to negotiate [remote work]. But for someone starting out or for someone at a lower level, even mid-level, um, it's going to be I find that it will be slightly difficult if we based on individual because there's a lot of trust.” (Participant 1, line 95) A participant reflects that although his current job and employment level afford him the ability to work remotely, this may not be possible for certain categories of work that require face-to-face interaction with the clients: “My job allows me to actually work completely remotely, you know. I'm not client-facing so I do not have to go into the office to see any clients.” (Participant 3, lines 310–312)

*Lack of employer support impacting an employee's capacity to work remotely* Reflects that where employers do not support the employee's individual needs nor understand the employee's limitations, negotiating engagement in remote work as a reasonable accommodation for persons with SCI can be challenging. Furthermore, participants indicated that remote work success was affected by an inflexible or micromanagement style	“You can have the best intentions, have the confidence, have the skills, but if the organisation is not open … for other people to understand your limitations and your situations, that could be very alienating for that individual.” (Participant 1, line 129) “It [remote work] was] very difficult ‘cause she was very, very, very micromanaging.” (Participant 3, line 230)

Theme four: intrinsic barriers of remote work Participants experienced that remote work (in particular if undertaken on a full time basis) decreased social engagement. This was due to employees working in individual areas or within their homes, with virtual contact being the main form of work interaction. This proved to be a barrier to the employee's success to work remotely. Participants reflected that the lack of social engagement lowered their motivation and occasionally made them feel despondent. This affected the quality of work. Moreover, participants experienced that remote work decreased their physical mobility as they were less mobile during the day	
*Remote work hampers social engagement* Reflects that engaging in remote work decreased the participants' social engagement. Participants indicated that office work provided opportunities to practice their social skills in a more structured environment. This specifically applied to those employees who were newly diagnosed with an SCI or employees entering the workplace for the first time. Working remotely does not afford employees this practice. Moreover, remote work can also impede work culture and employee morale. Lastly, remote work can decrease the visibility of persons with SCIs in the workplace	Decreased social engagement: “So I mean, I think the social aspect is quite a negative impact. You know, working from home ‘cause you do not have that social interaction with other people.” (Participant 3, line 147) “You do miss the interaction with fellow people.” (Participant 2, line 1797) “I would return back to work just for that reason… maybe once a week just to go chat to a few people … a few work friends …” (Participant 3, lines 1026–1029) A participant reflects that in-office work drove morale and motivation, whereas with remote work, it was difficult to validate colleagues virtually: “It also drives morale. It drives motivation in the workplace. It's not easy to validate someone across [virtual platforms] …” (Participant 2, Line 238-244) In-office work facilitated social engagement which serves as a confidence builder for persons with newly diagnosed SCI. In-office work, as compared to remote work, also has the potential to lessen stigma as coworkers have the opportunity to engage with persons with disabilities on a personal level: “I think it's actually good to get some office exposure ‘cause it forces … you going to present you think these people gonna think I'm in the wheelchair, you know? But then after a couple of years, you just think if I know my stuff. People then start seeing past that.” (Participant 4, lines 238–239). In-office work assists with the visibility of persons with SCIs: “You know it's (person's disability in the workplace) made visible to people.” (Participant 4, lines 286–288) (“It”, refers to a disability in the workplace. Therefore, the person with a disability is visible while engaging in-office work) “You know I always love when I see other people in wheelchairs and they dressed up.” (Participants 4, line 43)

*Subtheme 2: Remote work impacts physical mobility* Reflects that remote work engagement provides less opportunity for physical mobility, transfers as well as the need for wheelchair propulsion over long distances. Remote work thus exacerbates sedentary work	“Yeah, you definitely not as mobile as one should be. You know if you consider getting dressed … going to your car transferring into your car, driving to work … getting in the basement pushing around the office, you know pushing down to the lunch. Push into all the meeting rooms. You know the coffee stations.” (Participant 3, lines 1060–1070) “Moving around, you know, so I suppose that's one thing came to the office. You get more, well exercise, but these should be getting another call walking rolling to your office, getting some kind of blood flow.” (Participant 4, lines 447–448)

## Data Availability

The transcribed and audio-recorded interview data used to support the findings of this study have not been made available to protect participant confidentiality, as per restrictions by the Human Research Ethics Committee, Stellenbosch University.

## Appendix

### Interview Guide: English

Interview guide

Questions as follow:

Demographic information (interview 1)

- Name and surname of participant (allocation of pseudonym at this point)
- Interview date
- Gender
- Age
- SCI classification
- Type of occupation pre-SCI
- Type of occupation post-SCI

Questions (interview 1-3):

1. Tell me more about your current job?

*Probing questions*

- How long have you been at your current job?
- What did you do before your current job?

(2) Tell me more about where you work?
(3) Tell me how does a day at work look for you?
(4) How did your SCI impact your work?

*Possible probing questions:*

- Do you have the same job as before the SCI?
- How long have you been at your current job?

(5) Do you experience any difficulties of being a remote worker?

- Do you have sufficient work space to complete your job duties?
- Do you reside with people that affect your work habits?

(6) Do you feel remote working has helped you to perform your work?

- In what way has it benefitted you?
- What did you do before your current job?

(7) What has helped you to do your work remotely?
(8) What do you think could have been done differently or in addition to help you fulfil your remote work?

Do you have any last comments or suggestions?

Thank you for your time and taking part in the research study to discuss your work.

We will discuss further during the next interview where I will give you an opportunity to see how the data looks from our conversation today to ensure I understood your answers correctly.

The end.
